# Ferroptosis‐Related Genes Are Effective Markers for Diagnostic Targets of Crohn's Disease

**DOI:** 10.1002/iid3.70170

**Published:** 2025-03-14

**Authors:** Pengfei Liu, Qing Liu, Ye Tian, Pengpeng Cai, Jianan Bai

**Affiliations:** ^1^ Jiangyin People's Hospital Wuxi Jiangsu China; ^2^ The Fourth Affiliated Hospital of Nanjing Medical University Nanjing Jiangsu China; ^3^ The First Affiliated Hospital with Nanjing Medical University Nanjing Jiangsu China; ^4^ The Affiliated Sir Run Run Hospital of Nanjing Medical University Nanjing Jiangsu China

**Keywords:** Crohn's disease, diagnosis, ferroptosis, immune cells

## Abstract

**Introduction:**

Crohn's disease (CD) is a group of chronic transmural inflammation of gastrointestinal tract, which seriously harms the mental and physical health of adolescents. At present, there are still no specific markers that make the diagnosis of CD extremely difficult and poor prognosis. Iron deficiency is common in CD, yet the role of ferroptosis‐related genes in CD has not been elucidated.

**Methods:**

The serum iron and ferritin levels were detected in 107 newly diagnosed CD patients and 107 healthy volunteers in our hospital. Bioinformatics analysis was used to analyze the chip sequencing data of CD in GEO database. Immunohistochemical analysis of paired inflammatory and noninflammatory intestinal tissues from CD patients was performed to confirm the differential protein expression pattern of the target genes.

**Results:**

Patients with CD exhibited significantly reduced serum iron and ferritin levels compared to healthy controls. Transcriptomic analysis identified 40 upregulated and 31 downregulated ferroptosis‐associated genes in CD patients versus controls. LASSO regression and SVM‐RFE algorithms prioritized 13 hub genes (e.g., *CDKN2A*, *LCN2*, *STAT3*, *MT1G*), with a ROC curve demonstrating 100% specificity for combined biomarker analysis. Despite robust bioinformatic predictions, serum RNA levels of *CDKN2A*, *LIG3*, and *MTF1* showed no intergroup differences. Immuno‐reactivity score validated protein expression consistency for *LCN2, PANX1, LPIN1*, *PML*, *STAT3*, *PARP9*, *RELA*, *NEDD4*, and *MT1G* but not *PPARD* or *LCN2*. Expression patterns of these genes correlated with M0 macrophage infiltration, resting mast cells, and neutrophil recruitment, suggesting immune‐microenvironment interactions in CD progression.

**Conclusion:**

Combined detection of ferroptosis‐related genes is of great value in the diagnosis of CD.

## Graphical abstract

We found decreased serum iron and ferritin levels in CD patients compared to healthy controls. Combined detection of ferroptosis‐related genes is of great value in the diagnosis of CD, with the specificity even reaching 100%. The expression levels of these ferroptosis‐related genes are related to M0 macrophages, mast cells resting, and neutrophil recruitment.

## 1 Introduction

Crohn's disease (CD) represents chronic panmural inflammation of the gastrointestinal tract with segmental distribution, mostly found in the terminal ileum and adjacent colon. In Europe and Western countries, the annual incidence of CD is as high as 10.6–29.3/10,0000, among which the incidence of CD in children is 0.15–12/100,000. The prevalence rate even reaches 214–322/10,000 [1]. CD has the tendency of life‐long recurrence with poor prognosis, which brings a heavy burden to patients, families, and society [2, 3]. CD is still mainly diagnosed clinically, and differential diagnosis is difficult, with a high rate of misdiagnosis.

The destruction of the mucosal barrier caused by the abnormal death of intestinal epithelial cells (IECs) is an important pathogenesis of CD [4, 5]. Ferroptosis is a new type of iron‐dependent programmed cell death, which is driven by excessive accumulation of lipid peroxide catalyzed by free iron in cells [6, 7]. Ferroptosis has been proven to participate in many biological processes such as inflammation, tumor, and immunity and may become a drug therapy target [8, 9, 10, 11]. Previous studies have shown that iron deposition, glutathione depletion, glutathione peroxidase 4 (GPX4) inactivation, and lipid peroxidation are the basic characteristics of iron death in the damaged gastrointestinal tract of patients with inflammatory bowel disease [12]. GPX4 can inhibit lipid peroxidation [13, 14, 15]. IECs of patients with CD showed impaired activity of GPX4 [16]. The cytokine cascade of IECs triggered by arachidonic acid in the diet rich in omega‐6 polyunsaturated fatty acids is limited by GPX4, which will increase the risk of CD [16, 17].

Iron regulatory disorders and lipid peroxidation could be found in CD, and Ferrostatin‐1, an iron death inhibitor, could alleviate CD‐like colitis induced by 2, 4, 6‐trinitrophenol in mice [18]. Significant differences in the enrichment of ferroptosis‐related genes were observed between normal population and autoimmune disease samples, including CD, multiple sclerosis, systemic sclerosis‐related interstitial lung disease, and autoimmune orchitis [19]. However, the role of ferroptosis in CD is still unclear.

In this study, bioinformatics analysis was used to sort out the chip sequencing data of CD patients in the Gene Expression Omnibus (GEO) database. The expression of ferroptosis‐related genes was compared in CD patients and healthy controls. The diagnostic efficiency of combined detection of ferroptosis‐related genes for CD was explored.

## 2 Materials and Methods

### 2.1 Serum Iron and Ferritin Levels in CD Patients

At first, the serum iron and ferritin levels of 107 newly diagnosed, untreated, active CD patients (CD activity index more than 150) and 107 age‐ and gender‐matched healthy volunteers in our hospital were analyzed retrospectively.

### 2.2 Download, Annotation, and Correction of the CD Data Set

Then, we searched CD chip sequencing data matrix in the GEO database (https://www.ncbi.nlm.nih.gov/geo/). The mRNA expression was corrected by “Limma” and “BiocManager” packages with R software (https://www.r-project.org/). Package “ggord” was used to screen differentially expressed mRNA.

### 2.3 Differentially Expressed Ferroptosis‐Related Genes (DEFGs) in CD

To obtain a comprehensive set of genes related to ferroptosis, we downloaded the data set of marker genes, drivers, and suppressors of ferroptosis (http://www.zhounan.org/ferrdb/current/), and carried out Wilcox test with “Limma” package to analyze the difference of DEFGs. Finally, we exhibited the results with “pheatmap” package and analyzed the correlation with “Corrplot” package.

### 2.4 Enrichment Analysis of DEFGs

Gene ontology (GO) is often used for the enrichment analysis of gene functions, especially molecular functions (MF), biological pathways (BP), and cellular components (CC). Kyoto Encyclopedia of Genes and Genomes (KEGG) is often used to analyze the signaling pathways to which differential genes belong. The “enrichplot,” “ggplot2,” “circlize,” “RColorBrewer,” “dplyr,” “ComplexHeatmap,” and “clusterProfiler” packages were used to analyze the GO function and KEGG signal pathway of the DEFGs (*p* value filter = 0.05, *q* value filter = 1).

### 2.5 Screening of Hub Genes

Recurrent feature discrimination based on support vector machine (SVM‐RFE) has been proven as an effective machine learning technology for screening hub genes with an accuracy of over 85% [20]. In this study, we used “e1071” package for SVM‐RFE analysis (*n* fold=10). The lease absolute shrinkage and selection operator (LASSO) regression analysis has been used to calculate and select linear models and keep valuable variables with an accuracy of over 80% [21]. We employed “glmnet” package for LASSO analysis to obtain hub genes. Then, we employed “VENNDiagram” package to analyze the intersection of LASSO regression and SVM‐RFE as the most important hub genes.

### 2.6 Curve Analysis of Receiver Operating Characteristics (ROC)

The packages “glmnet” and “pROC” were used to create ROC curves to determine the area under the curve (AUC) for hub genes and evaluate their sensitivity for CD diagnosis.

### 2.7 Activity of Hub Genes Analyzed by Gene Set Enrichment Analysis (GSEA)

To observe the activated biological functions and signal pathways in PARP9 high‐expression and low‐expression groups, we used “Limma” package, “enrichplot” package, and “clusterProfiler” for GSEA analysis (*p* value cutoff = 1, *p* < 0.05).

### 2.8 Correlation Analysis of Hub Genes and Immune Cells

To investigate the various levels of infiltration of immune cell types between CD colitis and normal tissue, we employed “e1071” and “preprocessCore” packages (perm = 1000, *p*＜0.05). The “vioplot” package was used to observe the infiltration of immune cells in CD and control tissues with Wilcox test. To analyze the association between hub genes and immune cells, the “reshape2” package, “tidyverse” package “ggplot2” package were used to obtain the Spearman rank correlation coefficient.

### 2.9 Validation of the Hub Genes

The verification group chip matrix was annotated and corrected according to the above methods. The expression differences of hub genes were verified using “ppgubr” package.

### 2.10 Immunohistochemical Staining and Evaluation

Two‐step immunohistochemical detection of samples from 6 patients with CD and 6 healthy volunteers under colonoscopy was carried out according to the manufacturer's instructions. Immunohistochemical kit was purchased from Maixin Biotechnologies (Fuzhou, China). LCN2 (Abcam, ab216462, 1:2000), PML (Abcam, ab179466, 1:2000), PANX1 (Abcam, ab124969, 1:200), LPIN1 (Abcam, ab92316, 1:100), STAT3 (Abcam, ab68153, 1:200), PPARD (Abcam, ab137724, 1:100), PARP9 (Proteintech, 17535‐1‐AP, 1:50), MT1G (CusaBio, CSB‐PA17384A0Rb, 1:100), RELA (Proteintech, 10745‐1‐AP, 1:400) and NEDD4 (Proteintech, 21698‐1‐AP, 1:200) antibodies were used for incubation.

The expression of the above indexes was evaluated by immuno‐reactivity score (IRS): the positive rate (0, completely negative; 1, < 10% cells were positive; 2, 10%–50% cells were positive; 3, 51%–80% cells were positive; 4, > 80% cells were positive), and the staining intensity (0, completely negative; 1, weak; 2, moderate; 3, strong). The final score was multiplied by these two parts, which ranged from 0 to 12. IRS ≥ 1 was defined as positive.

### 2.11 Statistical Analysis

The statistical analyses of the data were conducted with R software. *p*＜0.05 was considered significant. The data with non‐parametric characteristics were analyzed by the Wilcoxon test. Correlation analysis was carried out by the Spearman correlation coefficient.

## 3 Results

### 3.1 Serum Iron and Ferritin Levels in Patients With CD

**Figure 1:**
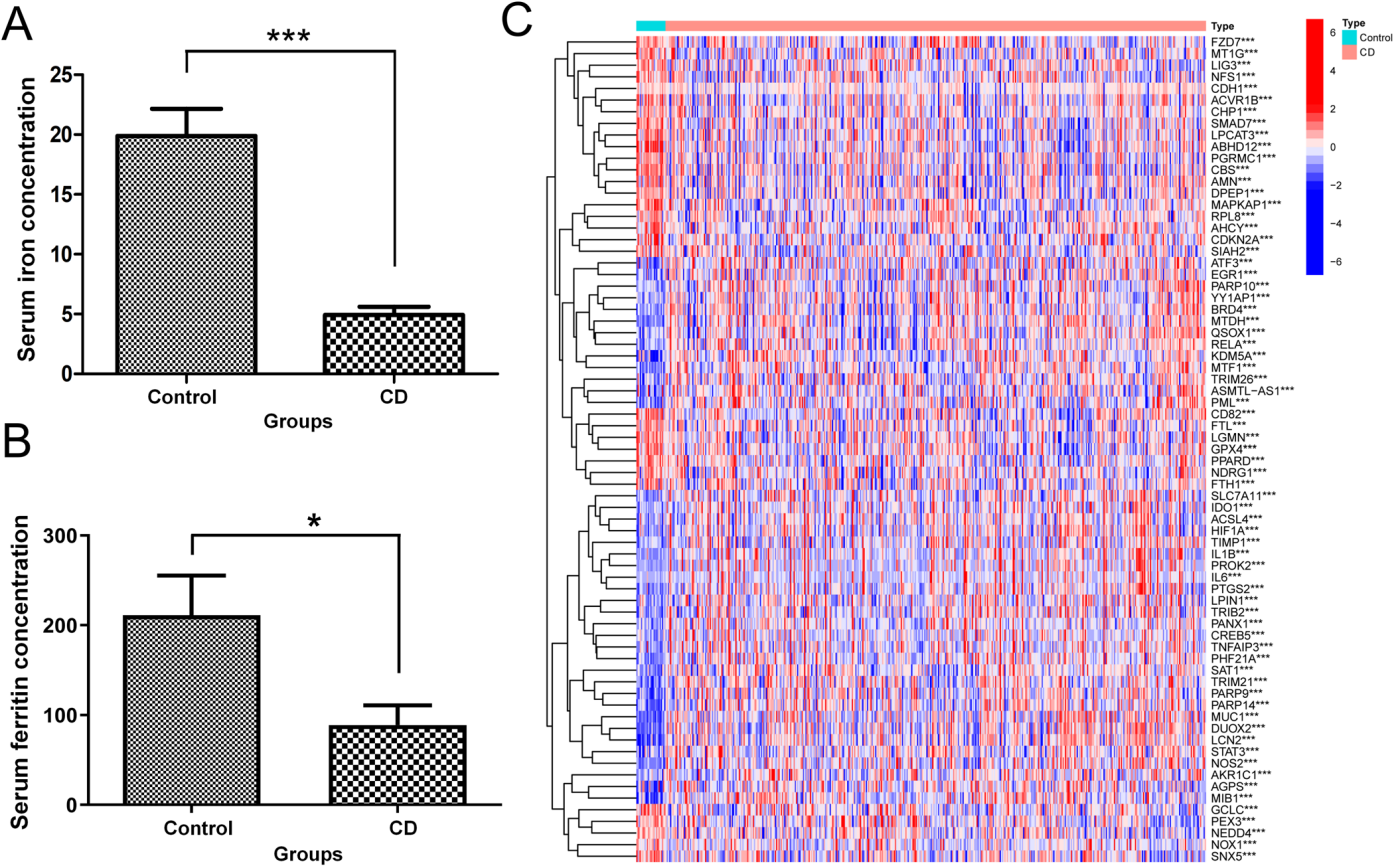
Differences in iron metabolism and ferroptosis between healthy and Crohn's disease (CD) patients. (A) The difference in serum iron level between the control group and CD patients. (B) The difference in serum ferritin level between the control group and CD patients. (C) Heatmap of differentially expressed ferroptosis‐related genes (DEFGs) between control group and CD patients. ***p＜0.001, *p＜0.05.

The average level of serum iron in CD patients was 4.93 μg/dL (reference range: 10.7–32.2 μg/dL), which was significantly lower than that in healthy volunteers (19.89 μg/dL, *p* < 0.0001) (Figure 1A). The average level of ferritin in CD patients was 88.78 ng/mL (reference range: 23.9–336.2 ng/mL), which was significantly lower than that in healthy volunteers (211.0 ng/mL, *p* < 0.05) (Figure 1B).

### 3.2 Data Characteristics of CD in GEO Database

We retrieved 92 CD chip data sets from GEO database and selected two chips with large sample size and complete expression data, among which GSE186582 was defined as the training group, including inflamed ileal mucosa samples of the surgical specimens (*n* = 200), ileal resection margins (*n* = 149) and neo‐terminal ileum 6 months after surgery (*n* = 122). Twenty‐five ileal mucosa samples from noninflammatory bowel disease controls (*n* = 25) were compared. The other chip of GSE179285 was defined as the validation group, including cases with Crohn's disease (*n* = 47) or normal healthy controls (*n* = 31) undergoing ileocolonoscopy (Figure 1C).

### 3.3 Expression of Ferroptosis‐Related Genes in CD Patients and Control Groups

A total of 513 genes related to ferroptosis, including driver genes (*n* = 264), suppressor genes (*n* = 238), and marker genes (*n* = 11) were obtained through an online search. The differential gene heat map related to ferroptosis between CD patients and healthy controls was obtained, among which 40 genes were upregulated and 31 genes were downregulated. Classical ferroptosis inhibitors such as GPX4, SLC7A11, and PPARD were downregulated clearly in CD group, indicating the increase of ferroptosis (Figure 1C).

### 3.4 Enrichment of Hub Genes

**Figure 2:**
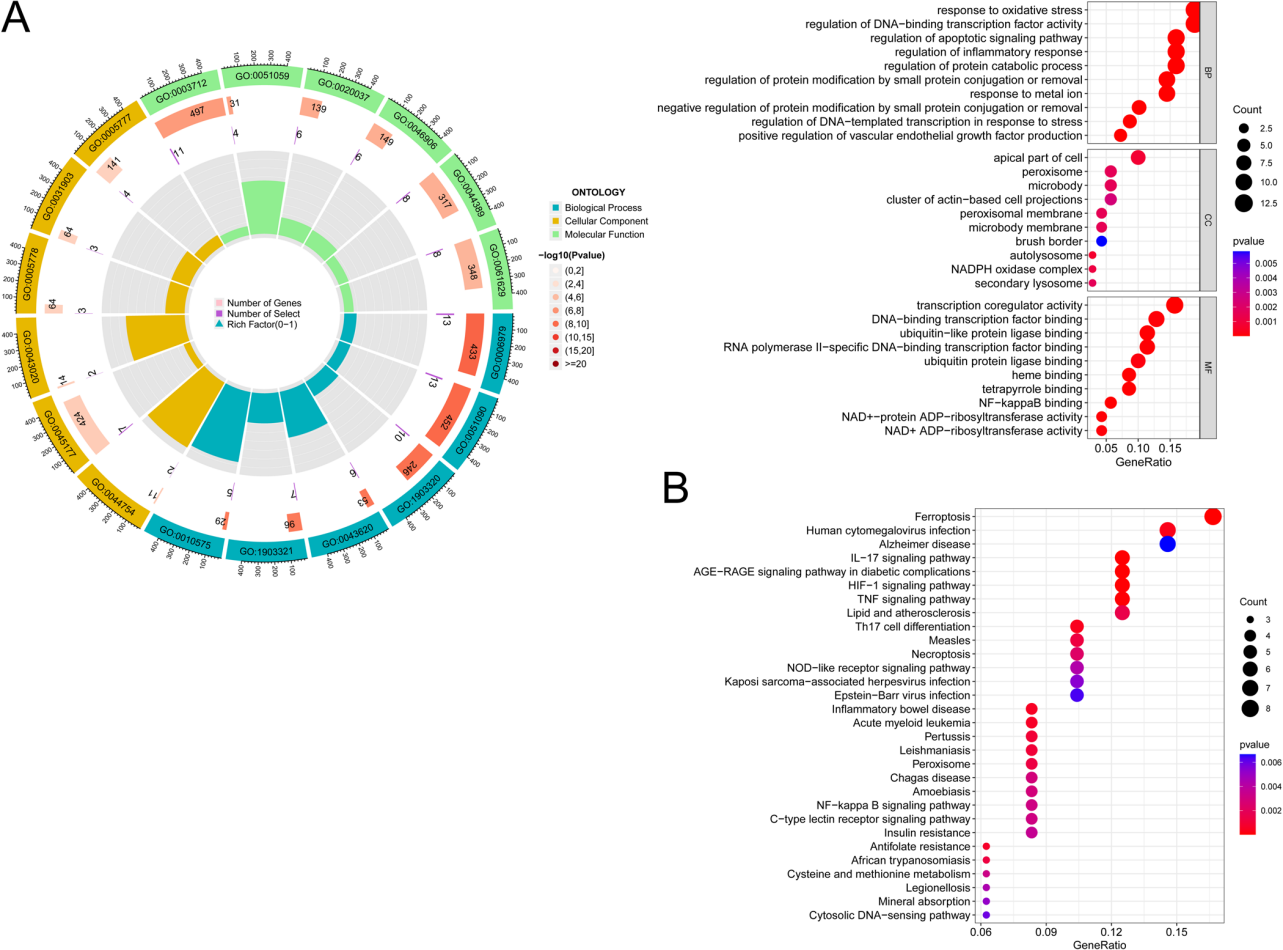
Gene ontology (GO) analysis and Kyoto Encyclopedia of Genes and Genomes (KEGG) analysis of differentially expressed ferroptosis‐related genes (DEFGs). (A) GO analysis of DEFGs. (B) KEGG analysis of DEFGs.

We carried out GO enrichment analysis on the above hub genes. For biological process (BP) enrichment, the signals of oxidative stress, apoptosis, and inflammatory reaction were surely enriched. Cellular component (CC) enrichment was related to the apical part of the cell, peroxisomes, and microbodies. Enriched molecular function (MF) was connected to transcription coregulator activity, DNA‐binding, transcription factor binding, and nuclear factor‐kappa B (NF‐κB) binding (Figure 2A). For KEGG analysis, ferroptosis signal, IL‐17 and TNF‐ɑ signal, and Th17 cell differentiation were indeed enriched (Figure 2B).

### 3.5 Screening of Hub Genes

**Figure 3:**
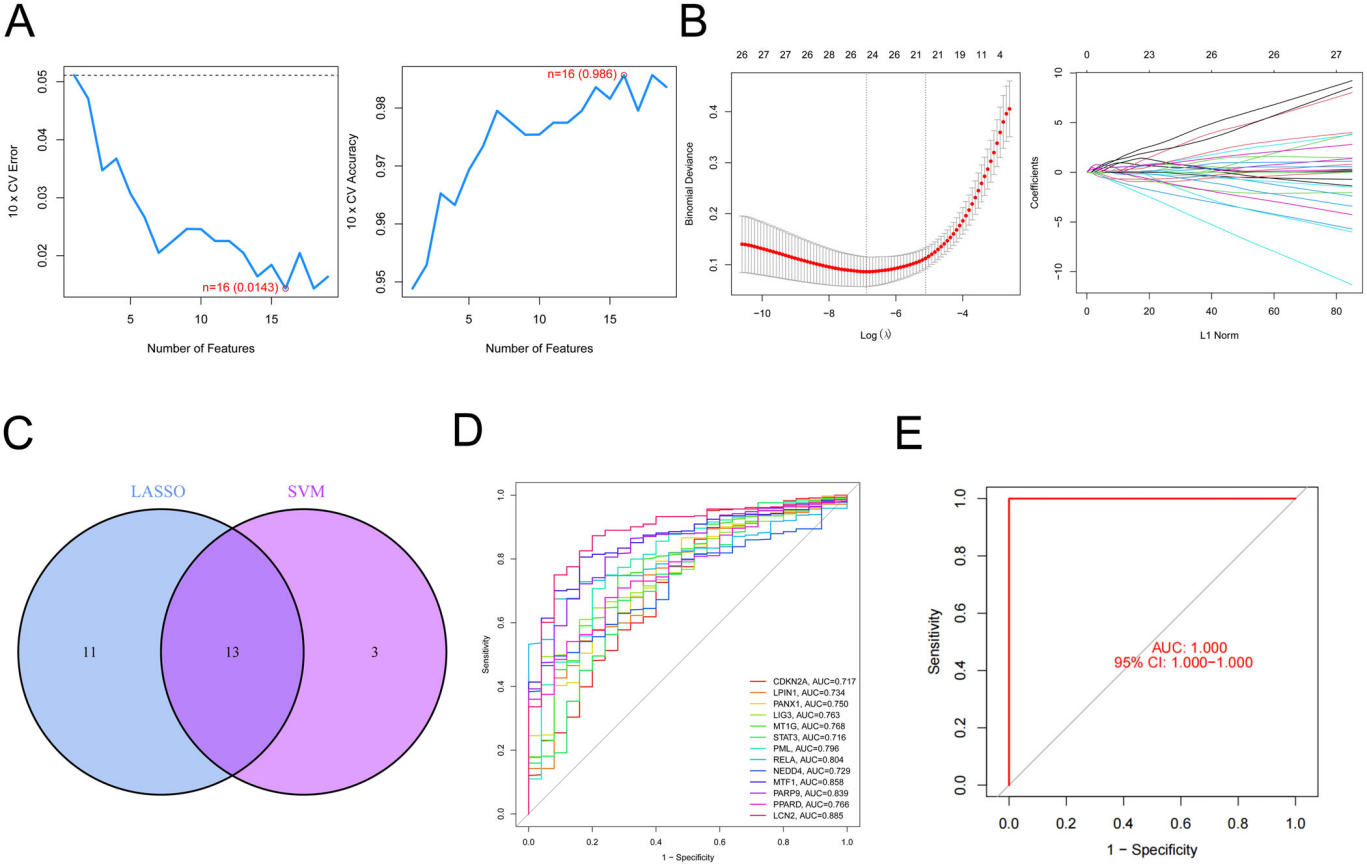
Screening of hub genes. (A) Lease absolute shrinkage and selection operator (LASSO) regression analysis revealed 24 characteristic genes related to ferroptosis. (B) Sixteen characteristic genes related to ferroptosis were screened by the recurrent feature discrimination based on the support vector machine (SVM‐RFE) method. (C) The intersection genes of LASSO regression analysis and SVM‐RFE were obtained by Wenn analysis. (D) The specificity of single hub gene in Crohn's disease (CD) diagnosis was observed by the receiver operating characteristics (ROC) curve. (E) The specificity of the combined detection of hub genes in CD diagnosis was observed by the ROC curve.

We used two machine algorithms to identify the hub gene: SVM‐RFE and LASSO regression analysis. Among them, 16 hub genes were screened by SVM‐RFE and 24 hub genes were screened by LASSO regression analysis, respectively (Figure 3A,B). Wenn analysis was performed with the results of the above two methods, and 13 intersecting hub genes were obtained (CDKN2A, LPIN1, PANX1, LIG3, MT1G, STAT3, PML, RELA, NEDD4, MTF1, PARP9, PPARD, LCN2) (Figure 3C).

### 3.6 The Specificity of Hub Gene for CD Diagnosis

To validate the sensitivity of CD diagnosis of the above hub genes, we used ROC analysis. CDKN2A (AUC: 0.717), LPIN1 (AUC: 0.734), PANX1 (AUC: 0.75), LIG3 (AUC: 0.763), MT1G (AUC: 0.768), STAT3 (AUC: 0.716), PML (AUC: 0.796), RELA (AUC: 0.804), NEDD4 (AUC: 0.729), MTF1 (AUC: 0.858), PARP9 (AUC: 0.839), PPARD (AUC: 0.766) and LCN2 (AUC: 0.885) were found to have high AUC values (Figure 3D). Interestingly, when these hub genes were jointly detected, the specificity of CD diagnosis reached 100% (Figure 3E).

### 3.7 Validation of Hub Genes

**Figure 4:**
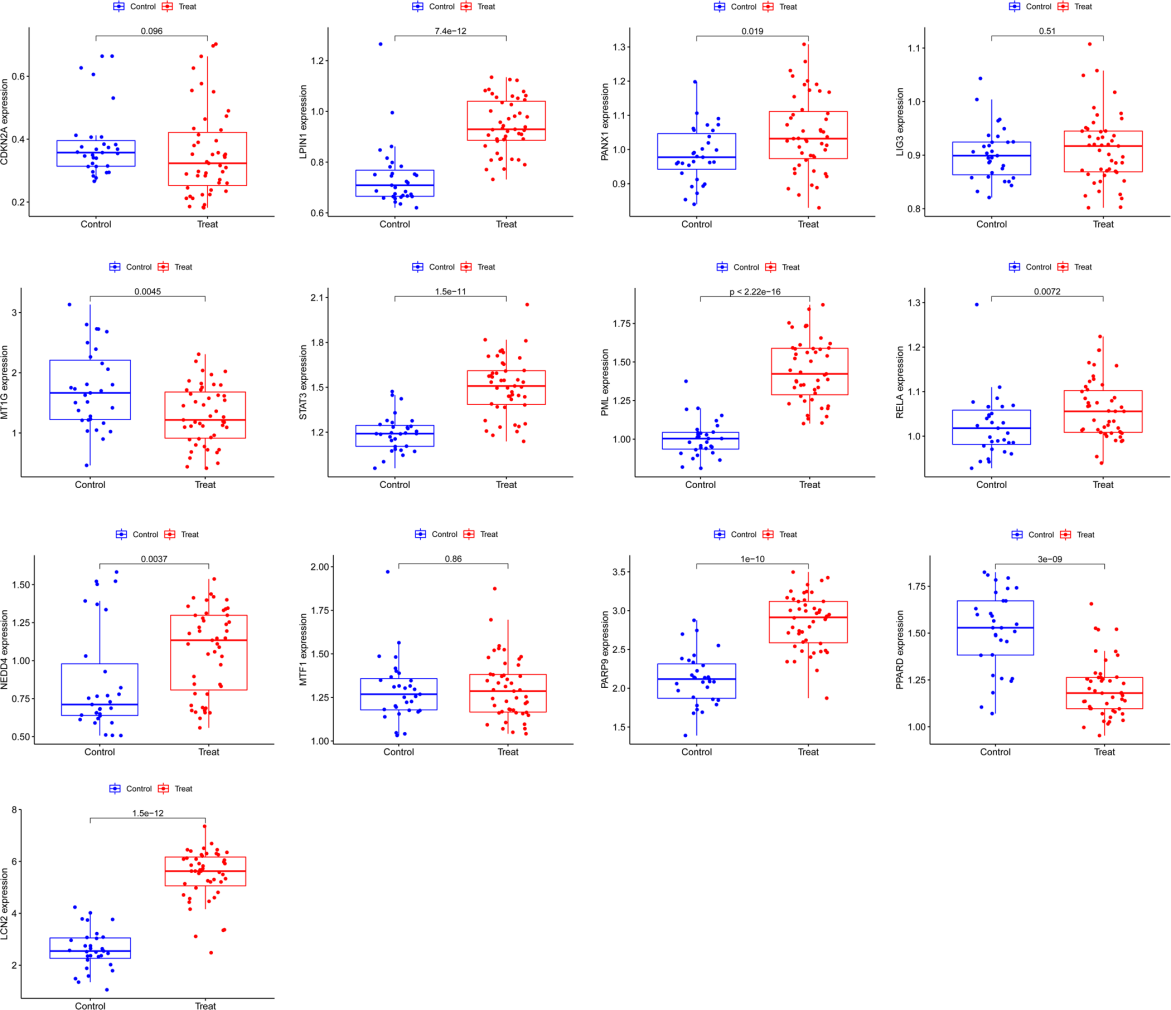
The expression of 13 hub genes was verified in the chip data set of verification group. Ten hub genes were confirmed as differential expression genes.

We confirmed the expression of the hub genes in CD using GSE179285 chip data and found that CDKN2A (*p* = 0.096), MT1G (*p* = 0.0045), and PPARD (*p* = 3 × 10^−9^) were downregulated in CD group than the control group. While LPIN1 (*p* = 7.4 × 10^−12^), PANX1 (*p* = 0.019), LIG3 (*p* = 0.51), STAT3 (*p* = 1.5 × 10^−11^), PML (*p*＜2.22 × 10^−16^), RELA (*p* = 0.0072), NEDD4 (*p* = 0.0037), MTF1 (*p* = 0.86), PARP9 (*p* = 1 × 10^−10^), and LCN2 (*p* = 1.5 × 10^−12^) were all upregulated in CD group than control group (Figure 4).

**Figure 5:**
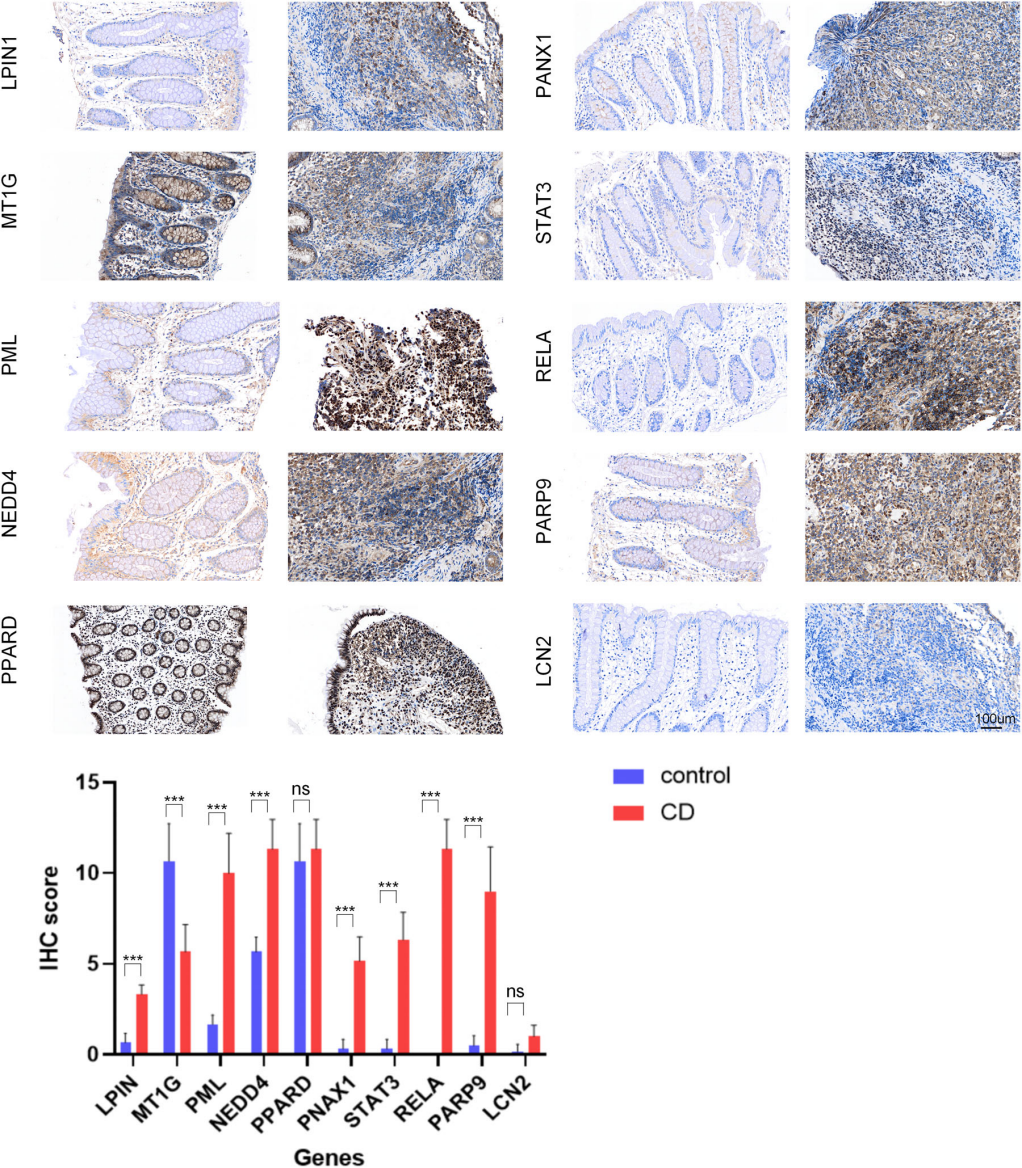
Immunohistochemical detection of 10 differential expression hub genes in inflammatory tissues and noninflammatory tissues of Crohn's disease (CD) patients. ***p＜0.001.

Compared with the noninflammatory tissues of CD patients, the upregulated proteins (LPIN1, PML, PANX 1, STAT3, PARP9, RELA, NEDD4) and downregulated protein (MT1G) in inflammatory tissues were confirmed according to IRS score, which was the same as the RNA expression in the serum. There was no significant difference in the expression level of PPARD and LCN2 between inflammatory tissues and noninflammatory tissues (Figure 5).

### 3.8 Infiltration of Immune Cells in CD Samples and Control Group From Chip Database

**Figure 6:**
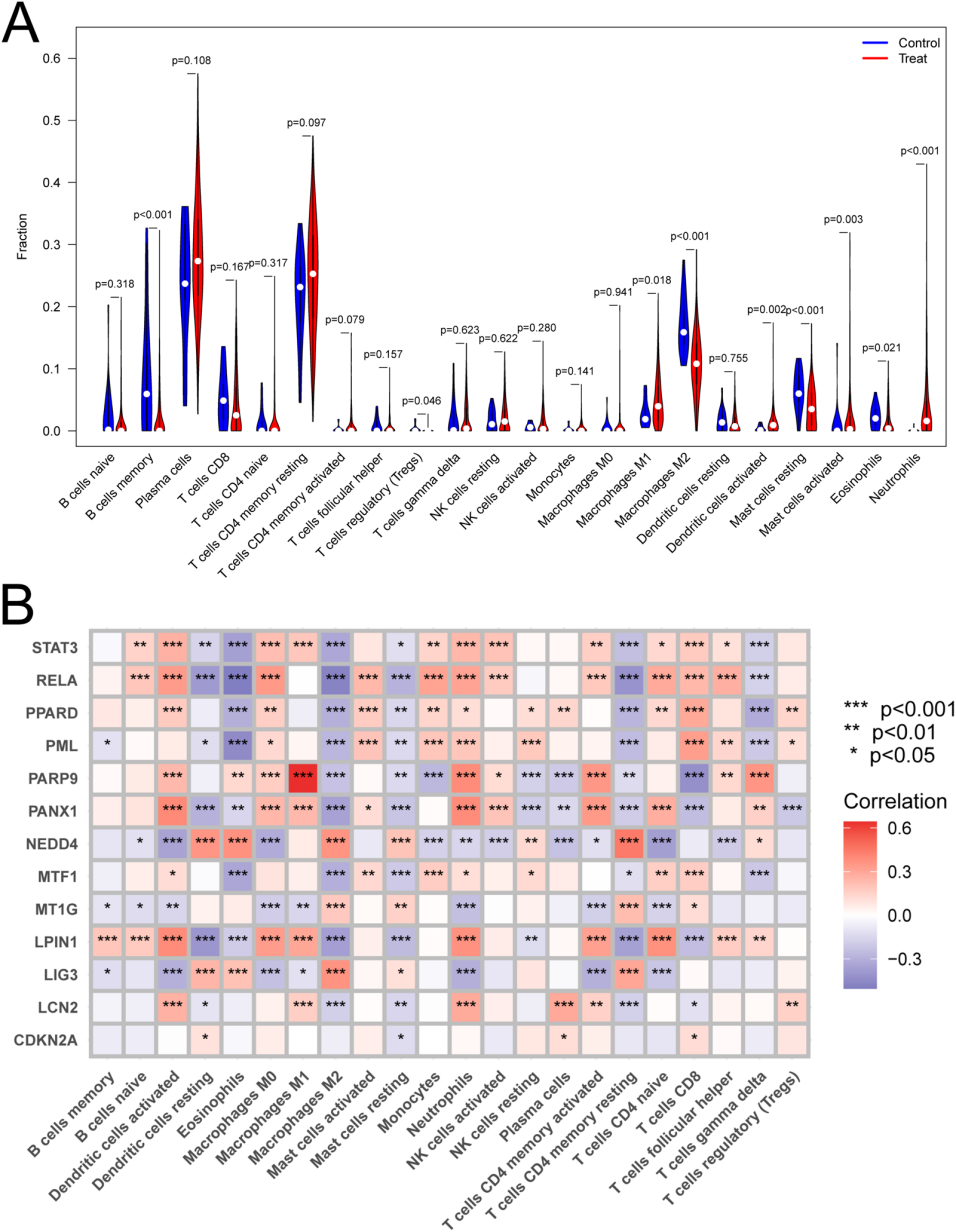
The relation of hub genes and immune regulation. (A) Analysis of immune cells infiltration in control group and Crohn's disease (CD) group. (B) Correlation analysis between hub genes and immune cells. CD group is served as a treat group. ***p＜0.001, **p＜0.01, *p＜0.05.

For the analysis of infiltration of immune cells of tissues of CD patients and healthy controls, we found that B cells memory, macrophages M2, mast cells resting and eosinophils were significantly less in CD group than in the normal group. Meanwhile, the plasma cells, macrophages M1, dendritic cells activated and neutrophils were indeed more in CD group than in the control group (Figure 6A).

### 3.9 Correlation Between Hub Genes and Immune Cells

To observe whether ferroptosis regulated the infiltration of immune cells in CD, we performed a correlation analysis between the hub genes and immune cells, suggesting that ferroptosis was involved in immune regulation. We found that the above hub genes were correlated with most immune cells. Some of them showed positive correlation, such as signal transducer and activator of transcript 3 (STAT3) and dense cells activated, while some of them showed positive correlation, such as v‐rel reticuloendotheliosis viral oncogene homolog A (RELA) and eosinophils (Figure 6B).

## 4 Discussion

The diagnosis and treatment of Crohn's disease are still facing great challenges. Traditional markers such as anti‐Saccharomyces cerevisiae antibodies (ASCA) and new serum markers such as anti‐flagellin antibodies and anti‐outer membrane porin C antibodies have certain diagnostic values, but their sensitivity and specificity are still poor. Lack of specific diagnostic markers and endoscopic manifestations leads to misdiagnosis in the clinic. Therefore, we still need to explore more effective diagnostic markers. Although some progress has been made in treatment, such as infliximab, ustekinumab, guselkumab, and so forth, which have achieved certain curative effects [22, 23, 24]. However, CD cannot be completely cured and tends to relapse for life, causing great harm to the body and mind of patients.

Cigarette smoking, infectious microbes, genetic susceptibility, and a dysregulated immune system can result in mucosal inflammation and death of IECs [25, 26]. The common ways of cell death are apoptosis, necrotic apoptosis, cell scorch, autophagy, and recently discovered ferroptosis and copper death [27, 28]. Ferroptosis is a new type of iron‐dependent programmed cell death discovered in 2012, which is driven by excessive accumulation of lipid peroxide catalyzed by free iron in cells [6]. During inflammation, serum ferritin tends to increase. However, we found that the serum iron and ferritin levels of CD patients were significantly lower than those of healthy people, indicating the iron metabolic disorder. The role and molecular mechanism of ferroptosis in the development of CD are still unclear.

By bioinformatics analysis, we sorted out the data sets of human CD‐related chips GSE186582 and GSE179285 in GEO database. A total of 71 DEGs were identified between CD patients and healthy controls. Some ferroptosis suppressors such as GPX4 were downregulated, which might lead to the occurrence of ferroptosis in intestinal epithelial cells. To find the hub genes among them, we employed two machine learning algorithms and intersected the results to obtain 13 hub genes.

ROC–AUC describes the true positive rate and false positive rate under different thresholds. The closer the value is to 1, the better the model performance. Matthews correlation coefficient is suitable for models with unbalanced categories. Accuracy is suitable for models with balanced categories. Precision focuses on reducing false positives. F1‐score focuses on balancing precision and recall. The joint diagnostic accuracy of our hub genes selected is close to 1, so we chose the ROC–AUC to evaluate the performance of the model. Through ROC curve, the AUC of these hub genes were all above 0.7, and the combined detection made the AUC reach 100%, which undoubtedly provided great help for the clinical diagnosis of CD. After other chip data and immunohistochemical verification, it was finally confirmed that the expression of nine genes (LCN2, PANX1, LPIN1, PML, STAT3, PARP9, RELA, NEDD4, and MT1G) in CD was significantly up/down regulated than the control group, which may provide the potential theoretical basis for developing clinical diagnostic markers of CD. Among them, the roles of LPIN1, PARP9, NEDD4, and MT1G in the development of CD have not been reported.

Then we analyzed the immune cells infiltrated in CD and found that B cells memory and macrophages M2 were significantly less in CD group, while plasma cells, macrographs M1, and neutrophils were more in CD group than in the control group, which was similar to the previous report [29, 30]. There was a significant correlation between ferroptosis‐related genes and immune cell infiltration, suggesting an important role of ferroptosis in immune regulation. Among these immune cells, activated dendritic cells, macrophages M2 and activated memory CD4^+^ T cells were related to all of the above 9 ferroptosis‐related genes.

Taken together, our results provide an ideal solution to the difficult situation of CD diagnosis and a potential target for the treatment of CD.

## Author Contributions

**Pengfei Liu:** data curation, resources, software, writing – original draft. **Qing Liu:** formal analysis, investigation, methodology, validation, writing – review and editing. **Ye Tian:** resources, supervision, validation, visualization, writing – review and editing. **Pengpeng Cai:** data curation, formal analysis, funding acquisition, investigation.

## Ethics Statement

This study was performed according to the Declaration of Helsinki and approved by the Ethics Committee of the First Affiliated Hospital of Nanjing Medical University.

## Conflicts of Interest

The authors declare no conflicts of interest.

## Data Availability

The relative experimental methods used to support the findings of this study are included within the article.
